# Spatial and Temporal Dynamics of Mass Mortalities in Oysters Is Influenced by Energetic Reserves and Food Quality

**DOI:** 10.1371/journal.pone.0088469

**Published:** 2014-02-14

**Authors:** Fabrice Pernet, Franck Lagarde, Nicolas Jeannée, Gaetan Daigle, Jean Barret, Patrik Le Gall, Claudie Quere, Emmanuelle Roque D’orbcastel

**Affiliations:** 1 Ifremer, Laboratoire Environnement Ressource du Languedoc Roussillon, Bd Jean Monnet, Sète, France; 2 UMR LEMAR Ifremer/CNRS/UBO/IRD, Technopole de Brest-Iroise, Plouzané, France; 3 Geovariances, Avon, France; 4 Université Laval, Département de mathématiques et de statistique, Pavillon Alexandre-Vachon, Québec, Québec, Canada; Université Catholique de Louvain, Belgium

## Abstract

Although spatial studies of diseases on land have a long history, far fewer have been made on aquatic diseases. Here, we present the first large-scale, high-resolution spatial and temporal representation of a mass mortality phenomenon cause by the Ostreid herpesvirus (OsHV-1) that has affected oysters (*Crassostrea gigas*) every year since 2008, in relation to their energetic reserves and the quality of their food. Disease mortality was investigated in healthy oysters deployed at 106 locations in the Thau Mediterranean lagoon before the start of the epizootic in spring 2011. We found that disease mortality of oysters showed strong spatial dependence clearly reflecting the epizootic process of local transmission. Disease initiated inside oyster farms spread rapidly beyond these areas. Local differences in energetic condition of oysters, partly driven by variation in food quality, played a significant role in the spatial and temporal dynamics of disease mortality. In particular, the relative contribution of diatoms to the diet of oysters was positively correlated with their energetic reserves, which in turn decreased the risk of disease mortality.

## Introduction

Since the mid-1970s, large-scale episodic events such as disease epidemics, mass mortalities, harmful algal blooms and other population explosions have been occurring in marine environments at a historically unprecedented rate [1]–[3]. Bivalve farming, because it relies directly upon natural marine environments and feeding resources, is presently faced with a number of severe risks and limiting factors. Historically, infectious diseases have seriously affected the marine bivalve industry. In the early 1970s, the French oyster industry suffered a serious crisis when irido-like virus infections decimated the Portuguese oyster, *Crassostrea angulata*, in European Atlantic waters [4]. The Pacific oyster *C. gigas* has since been introduced for culture along the coasts of France. Episodes of Pacific oyster mortality have been occurring for more than five decades in the major oyster-producing countries [5]. Most of these episodes have been classified as “summer mortality”, typically affecting animals during the warmer months of the year. Since 2008, massive mortality events in *C. gigas* oysters have been reported in almost all farming areas in France when seawater temperature exceeds 16°C [6]–[8]. These mortality events have been attributed to a combination of adverse environmental factors combined with the presence of ostreid herpes virus 1 (OsHV-1) [7]–[10]. Evidence suggests that OsHV-1 infection is a necessary cause, and a particular genotype, named OsHV-1 µvar [11], appears to have been the dominant viral genotype in the most serious mortality events of 2008–2011. Oyster mortalities were considerable in this period, particularly among seed stocks [6]–[8]. The devastation of oyster beds in France observed every year since 2008 has now resulted in a shortfall in shellfish supplies corresponding to approximately 40% of commercial production.

Although mass mortalities of oysters are clearly related to infectious diseases, they can also reflect an unfavourable energetic balance. In 2008, oysters in Mediterranean Thau lagoon (France) showed a sharp decrease in carbohydrate concentration and arrested accumulation of lipid reserves before mortalities occurred [10]. It was suggested that young oysters go through a phase of energetic weakness during the spring that renders them more susceptible to pathogen infections. Correspondingly, oysters with high energy reserves are probably less affected by pathogen infections than those with low levels [7], as reported for vertebrate species [12]–[15]. It is therefore likely that differences in energetic condition of oysters have a significant influence on the spatial and temporal dynamics of disease mortality. Given that the energetic condition of marine bivalves varies depending on food quality and availability [16]–[18], these parameters may also be related to the disease mortality of oysters. Although the quality and availability of food consumed by bivalves have drawn little attention in terms of host–parasite interactions [19], they have been demonstrated to modulate defense related mechanisms [18], [20]–[22].

The first objective of this paper is to explore the spatial pattern of disease mortality in oysters *C. gigas*. For instance, describing the distribution and dynamics of infectious disease is important for management [23], [24]. The spatial distribution of a disease is often examined by applying statistical methods to data collected during disease surveillance and then generating a map that describes spatial variation in risk. However, this task is often complicated by heterogeneities in the population of interest [14]. For example, in oysters, susceptibility to the disease varies with age, origin of oysters, farming practices, and life history traits [7]. These heterogeneities, if not accounted for, may obscure observation of important disease trends that may be of interest to resource managers. In order to circumvent this issue, disease mortality of oysters was investigated in susceptible animals deployed at 106 locations in the Thau lagoon, on the French Mediterranean coast, before the start of the annual epizootic event. Therefore, variance in disease mortality in these “sentinel oysters” reflects the location itself, while minimizing confounding effects. Disease mortality is expected to show strong spatial and temporal dependence due to the epidemic process of local transmission. Although terrestrial studies on the spatial aspects of diseases have a long history, driven by a necessity to understand diseases of humans, crops, farm animals and wildlife [25], spatial studies of aquatic diseases are much less frequent and generally focused on distribution of macro-parasites [26]–[33].

The second objective is to examine the relationships between energetic condition, food quality and the probability of disease mortality in oysters. Our hypothesis is that the spatial and temporal dynamics of disease mortality in oysters are partly driven by local differences in energetic reserves and food quality. To test this hypothesis, the probability of disease mortality of oysters was analysed in relation to their energetic reserves and the fatty acid composition of their neutral lipids. Marine bacteria, diatoms, dinoflagellates, terrestrial inputs and vascular plants show different combinations of specific fatty acids. Given that fatty acids are incorporated largely unaltered into the reserve lipids of primary consumers like oysters, they generally reflect the fatty acid profiles of the food consumed and therefore reveal useful information about trophic sources [34], [35].

## Materials and Methods

### Animals

Diploid oysters were produced at the Ifremer hatchery in La Tremblade in July 2010 and then maintained in the nursery at the Ifremer marine station in Bouin from August 2010 onwards. These oysters were maintained free of disease mortality from hatching until deployment in Thau lagoon on 15 March 2011. Mean shell length was 10 mm, mass was 0.7 g and age of oysters was 8 mo. These oysters had shown no disease mortality prior to deployment and OsHV-1 DNA was not detected in their tissues.

### Study Area

**Figure 1:**
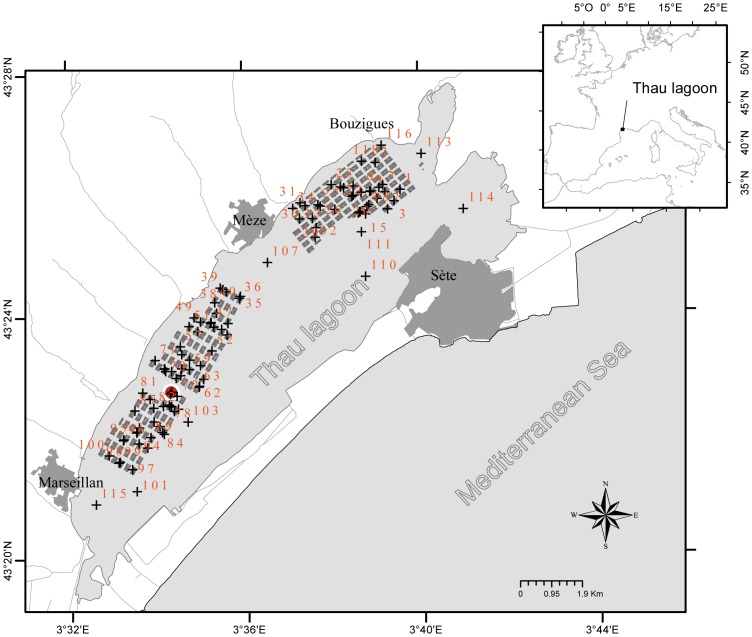
Sampling sites (black crosses along with non-continuous numbering) located in the Thau lagoon. Areas with grey boxes: bivalve farms; red circle: temperature probe.

The Thau lagoon on the French Mediterranean coast is an oyster farming area that accounts for about 10% of Pacific oyster production in France. Oyster growth rates in the lagoon are among the highest in France for this species. Thau lagoon is 19 km long, 4.5 km wide and 5 m deep on average (Figure 1). Shellfish are cultured in 3 areas of the lagoon, namely Bouzigues, Mèze and Marseillan, covering about 20% of the total surface area. The lagoon is almost completely closed off from the Mediterranean Sea, with only narrow connections through the Sète channel; other connections being negligible in terms of water exchange.

### Experimental Design

The oysters were placed in 106 pearl nets at an initial density of 150 individuals per pearl net, as commonly practised by oyster farmers. These pearl nets were deployed within the Thau lagoon in the farming areas of Bouzigues (n = 35), Mèze (n = 34) and Marseillan (n = 29) and also outside of the farming areas (n = 8). The sampling design was primarily developed to capture spatial and temporal trends within the farming areas. Fifteen additional sampling stations outside farming areas were added to allow capturing spatial trends at the lagoon scale, although only eight were maintained the duration of the study to produce useful data (Figure 1). The distribution of sampling stations corresponds to a random coverage of shellfish farms. Permission for deploying oysters outside of farming areas was issued by the French Ministry of Ecology and Sustainable Development, dept. of maritime affairs, in February 2011. For locations within farming areas, the owner of the farm gave permission to conduct the study on this site. The present field studies did not involve endangered or protected species.

**Figure 2:**
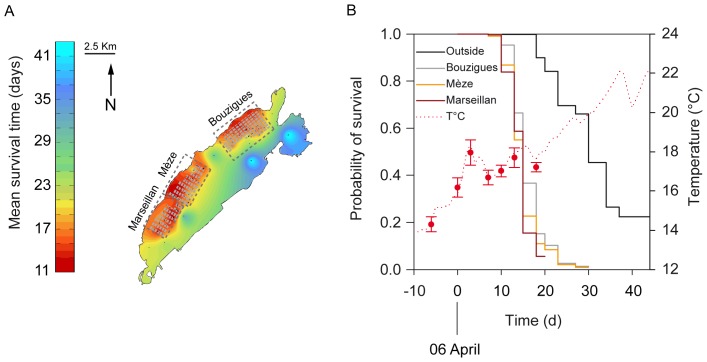
Survival of oysters in the Thau lagoon. (A) Kriged map of the mean survival time of oysters in the Thau Mediterranean lagoon. Mean survival time was measured as days from 6 April, when seawater temperature reached 16°C. Black points represent sampling sites, areas with grey boxes represent bivalve farms and dashed rectangles correspond to the three farming areas. (B) Left axis: survival functions of oysters for each area of the lagoon. Right axis: evolution of seawater temperature during the period of study. Temperature was recorded continuously at one location within the bivalve farming area of Marseillan (dotted line), and punctually at all locations (red circles, data are means ± SD).

Live and dead oysters were counted in each pearl net on 31 March; 6, 9, 13, 16, 19, 21, 24, 26 and 29 April; 3, 6, 10, 13 and 20 May 2011. Ten live oysters were randomly sampled from each pearl net on 6 and 16 April for laboratory analyses. Soft tissues of oysters were carefully removed from the shells, then pooled together and dipped into liquid nitrogen immediately after sampling before being stored at −80°C. These two sampling dates corresponded (1) to the time of the year when seawater temperature attains 16°C, a threshold temperature above which disease transmission is optimal and mortalities occur [6]–[8], [36], and (2) to the onset of the mass mortality, respectively (Figure 2).

Temperature and salinity (data not shown) were recorded every 10 min during the entire period of study, using autonomous CTD multiparameter recorders (NKE Instrumentation) deployed at Marseillan, within the bivalve farming area (43°22′44.86″ N, 3°34′15.88″ W, Figure 1). Temperature and salinity were also measured at each location for each sampling date using a WTW ProfiLab LF597-5 salinometer (Sigma-Aldrich, Lyon, France) to obtain information for the entire lagoon. Periodic temperature measurements taken at each location fit with the continuous temperature profile recorded at Marseillan (Figure 2). Overall, spatial variation in seawater temperature was ±0.4°C in Thau lagoon.

### Laboratory Analyses

Pooled oysters (n = 10 individuals) were ground with a MM400 homogeniser (Retsch) under liquid nitrogen, and the resulting powder was stored at −80°C and sub-sampled for pathogen detection and biochemical analyses.

Pathogen and energetic analyses were conducted as previously described ( [7], [10] and see File S1). Briefly, the detection and quantification of OsHV-1 DNA was carried out using a previously published real-time PCR protocol [37]. The energetic reserves triacylglycerol and carbohydrate were expressed in mg g^−1^ dry mass of tissues. The relative variation of these energetic reserves was calculated between 6 and 16 April according to the following formula:
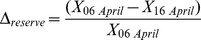
where *X* represents triacylglycerol or carbohydrate.

The fatty acid markers investigated in this study were the ratio of 16∶1n−7/16∶0 and 20∶5n−3, both of which indicate the contribution of diatoms to the diet of oysters and mussels; the sum of 18∶2n−6 and 18∶3n−3, which is generally considered as a marker of terrestrial inputs; the ratio of 18∶1n-9/18-1n-7, which is generally used as an indicator of carnivory; the ratio of polyunsaturated/saturated fatty acid (PUFA/SFA), which is an indicator of freshness; and the sum of iso- and anteiso-branched chain fatty acids and unbranched 15∶0 and 17∶0, which reflects the contribution of bacteria to the organic matter. These fatty acid food web markers are extensively used in trophic ecology [34], [35].

### Statistical Analyses

Nonparametric estimates of the survivor function were computed by the Kaplan–Meier method [38]. Survival time was measured as days from 6 April (time origin), when seawater temperature reached 16°C. The data were read as the number of dead animals within each experimental unit at each time interval for 13 intervals. Sampling areas were used as strata and the resulting survival estimates were compared by using the log-rank test of homogeneity of strata.

Two-way mixed model ANOVAs were conducted to determine potential differences in energetic reserves of oysters according to sampling area (Bouzigues, Mèze, Marseillan, outside) and time (6 and 16 April 2011). The term ‘Time’ was a random factor with two levels of repeated measurements. One-way ANOVAs were conducted to determine potential differences in fatty acid food web markers of oysters according to sampling area. Where differences were detected, Fisher’s protected LSD multiple comparison tests were used to determine which means were significantly different. This procedure controls the familywise error rate since those tests are only applied following a significant effect in the ANOVA table.

Detection frequencies of OsHV-1 DNA in oysters sampled on 6 April were analysed by chi-square tests of independence according to area (4 areas×2 outcomes [not detected/detected]).

The survival time curves of oysters in different sampling areas were compared using the Cox proportional hazards regression model [39], after adjustment for the effect of some static covariates like whether or not OsHV-1 DNA is detected, the levels of triacylglycerol and carbohydrate measured on 6 April, and the relative variation of these energetic reserves between 6 and 16 April. The tests on regression parameters were made using the robust sandwich method [40]. The proportionality of hazards was checked by testing the interaction between time and levels of treatment (areas).

Multiple regression models were used to examine the relationship between energetic reserves and food web fatty acid markers of oysters measured on 6 April. Analyses were done on the best transformation of response variables among the Box-Cox family [41]. Triacylglycerol and carbohydrate were square root transformed while Δ triacylglycerol and Δ carbohydrate were log transformed to eliminate skewness and to reach normality. For each model, explanatory variables (fatty acid food web markers), including cross-product terms and interactions, were selected based on the Akaike information criteria (AIC) in a stepwise method. This means that explanatory variables were added into the model one by one, by selecting at each step the one that minimizes the AIC criteria. This was done until the stopping rule was met, which consists of the smallest residual sum of squares using the leave-one-out cross-validation technique.

These statistical analyses were conducted using LIFETEST, FREQ, MIXED, PHREG, CORR, REG and GLMSELECT procedures of the SAS software package (SAS 9.3, SAS institute, Carry, USA).

Spatial structures of the mean survival time, energetic reserves and trophic markers of oysters were described through variograms, allowing quantification of the spatial dependency and its partitioning among distance classes [42]. Statistical models (linear, exponential and spherical) were fitted to the variograms to produce interpolated maps by kriging for each variable using ISATIS [43].

## Results

### Disease Mortality of Oysters

The sentinel oysters deployed in Thau lagoon were severely hit by the mass mortality phenomenon. Final survival was lower than 15% in 104 out of 106 experimental units (Figure S2.1 in File S2). Survival was almost 100% in the two remaining experimental units located in the south-eastern extremity of the lagoon (Figure S2.1 in File S2).

Survival time was computed in days from 6 April, when seawater temperature reached 16°C, and used for spatial analyses (Figure 2A). Survival time of oysters varied from 8.9 d to 45.0 d, the latter corresponding to the entire duration of the experiment. Survival time of oysters showed strong spatial dependence (Figure S2.2 in File S2). Survival functions of oysters differed among areas (Figure 2B, log-rank test *p*<0.001). Mean survival time of oysters was particularly low within the bivalve farming areas (16.0 d ±0.06, 14.9 d ±0.06, and 14.1 d ±0.04 at Bouzigues, Mèze and Marseillan, respectively) compared to that of animals deployed outside the farming areas where it reached 29.7 d ±0.29.

On 6 April, when seawater temperature attained 16°C, OsHV-1 DNA was detected in 33 experimental units out of 104 (Table 1, Figure S3.1 in File S3). There was no significant effect of sampling areas on the detection of OsHV-1 DNA in oysters (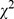 test, p = 0.095, Figure S3.1 in File S3). On 16 April, when the mass mortality event started (Figure 2B), OsHV-1 DNA was detected in all experimental units (Table 1). Therefore, the mass mortality of oysters coincided with the general spread of OsHV-1 in oysters of the Thau lagoon.

**Table 1:** Detection of OsHV-1 DNA according to areas and time in oysters deployed in the Thau lagoon.

Areas	OsHV-1 DNA					
	6 April		∑	16 April		∑
	Not detected	detected		not detected	detected	
Within farming areas	66	30	96	0	92	92
Bouzigues	23	12	35	0	30	30
Mèze	27	5	32	0	33	33
Marseillan	16	13	29	0	29	29
Outside farming areas	5	3	8	0	8	8
∑	71	33	104	0	100	100

Data are number of pearl nets containing the sampled oysters (n = 10 pooled animals for each analysis).

### Energetic Reserves

**Figure 3:**
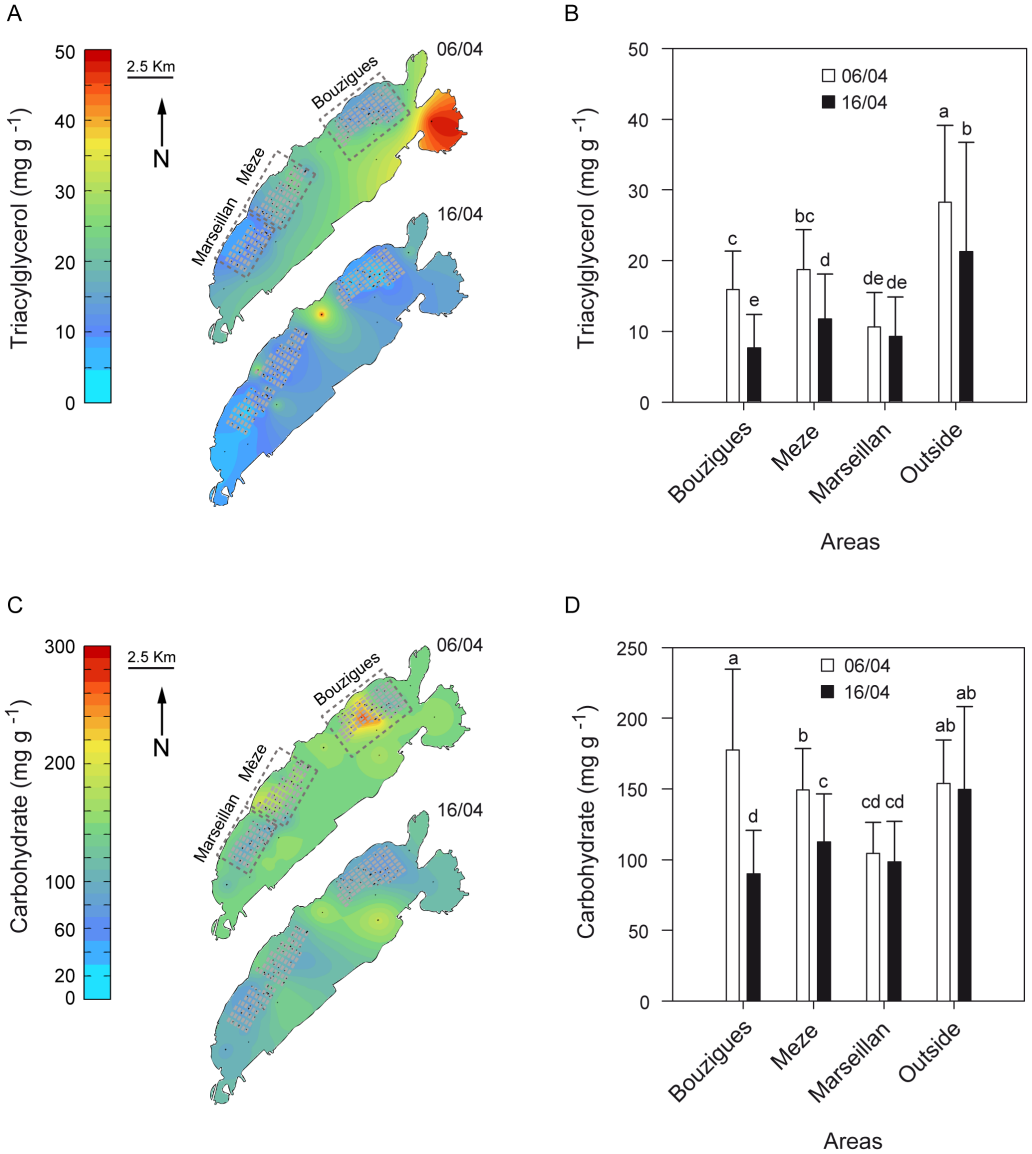
Energetic reserves of oysters in the Thau lagoon. (A, C) Kriged maps of the triacylglycerol and carbohydrate levels in oysters in the Mediterranean Thau lagoon measured on 6 April when seawater temperature reached 16°C, and 16 April at the beginning of the mass mortality phenomenon. Black points represent sampling sites, areas with grey boxes represent individual bivalve farms and dashed rectangles correspond to the three farming areas. (B, D) Triacylglycerol and carbohydrate levels of oysters as a function of time and area in the Thau lagoon. Letters indicate significant differences. Data are means ± SD.

Level of triacylglycerols in oysters sampled on 6 April varied markedly, from 2.0 mg g^−1^ wet tissues to 53.5 mg g^−1^, depending on location in the lagoon (Figure 3A). On average, the level of triacylglycerol in oysters maintained outside the farming areas was 1.5, 1.8 and 2.7 times higher than the levels of those held in the farming areas of Mèze, Bouzigues and Marseillan, respectively (Figure 3B). Ten days later, at the onset of the mass mortality event, levels of triacylglycerol dropped by 49%, 36% and 29% in oysters held at Bouzigues, Mèze and outside of the farming areas, respectively (Figures 3A, B). The level of triacylglycerol in oysters held outside the farming areas remained higher than that of oysters maintained within the farming areas. The level of triacylglycerol in oysters held at Marseillan remained low and did not change significantly over the sampling time.

Level of carbohydrates in oysters sampled on 6 April varied depending on location in the lagoon (Figure 3C) and had dropped by 51% and 19% at Bouzigues and Mèze, respectively, by 16 April (Figures 3C, D). Also, the level of carbohydrates in oysters held outside the farming areas remained higher than that of oysters maintained within the farming areas on 16 April. As observed for triacylglycerol, the level of carbohydrate in oysters held at Marseillan remained low and did not change significantly between sampling times (Figures 3C, D).

Levels of triacylglycerols and carbohydrates in OsHV-1 positive oysters on 6 April were slightly lower or similar to levels in OsHV-1 negative animals (Triacylglycerol: 14.2 *vs*. 17.4 mg g^−1^ wet tissues, p = 0.047, Carbohydrate: 149.6 vs. 139.6 mg g^−1^ wet tissues, p = 0.337).

### Proportional Hazards Model

Table 2 shows the final model describing the effects of sampling areas, detection of OsHV-1 DNA and energetic reserves on the risk of mortality in oysters (hazard ratio). The risk of oyster mortality was 9.6 to 14.9 times higher in the farming areas of the Thau lagoon than outside these areas (Table 2). The risk of mortality of oysters was similar among the three farming areas (p>0.05). Risk of mortality was 1.4 times higher in oysters when OsHV-1 DNA was detected on April 6, than when it was not. Although a relation between the risk of mortality and energetic reserves of oysters measured on April 6 was not significant, survival time increased with difference of energetic reserves between 6 and 16 April (Table 2). Therefore, the capacity of oysters to use their energetic reserves during the infection period was associated with a lower risk of mortality. It is, however, noteworthy that energetic reserves of oysters measured on April 6 were correlated with their utilisation between 6 and 16 April (r values were 0.377 and 0.511 for triacylglycerols and carbohydrates, respectively, p<0.001).

**Table 2:** Analysis of the effect of covariates on survival of oysters in the Thau lagoon by a Cox proportional hazards model.

Covariate	Hazardratio	Hazardratio SE	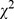	p value
Areas	n.a	n.a	33.2	<0.001
Bouzigues vs Outside	9.671	4.282	26.3	<0.001
Mèze vs outside	14.870	6.975	33.1	<0.001
Marseillan vs outside	13.551	6.876	26.4	<0.001
Marseillan vs Mèze	0.911	0.228	0.1	0.710
Marseillan vs Bouzigues	1.401	0.870	1.9	0.165
Mèze vs Bouzigues	1.537	0.346	3.7	0.056
OsHV-1 DNA	1.405	0.209	5.2	0.022
Triacylglycerol	0.971	0.018	2.4	0.119
Carbohydrate	1.004	0.002	3.3	0.070
Δ Triacylglycerol	0.771	0.082	6.0	0.014
Δ Carbohydrate	0.680	0.108	5.9	0.015

For each covariate, the following elements are provided: its parameter estimate and the corresponding instantaneous hazard ratio and its standard error (SE); the χ^2^ statistic (with 1 degree of freedom for all tests except for areas where it was 3) and the resulting p value for the type II test from the complete model. Covariates are the presence or not of OsHV-1 DNA in oysters (on 6 April), their triacylglycerol and carbohydrate reserves (on 6 April), and the relative variation of these energetic reserves (Δ) between 6 and 16 April.

### Fatty Acid Food Web Markers

Kriged maps of fatty acid food web markers show high spatial variation for diatom (16∶1n-7/16∶0 and 20∶5n-3), terrestrial (18∶2n-6+18∶3n-3), animal (18∶1n-9/18∶1n-7) and bacterial contributions to the diet of oysters (Figure S4.1 in File S4).

Multiple regression models showed that the two diatom markers, 16∶1n-7/16∶0 and 20∶5n-3, explained 28.3% of the spatial variance in triacylglycerol levels of oysters, whereas 20∶5n-3 and PUFA/SFA explained 41.3% of the spatial variance in carbohydrate (Table 3). The diatom marker 20∶5n-3 explained the largest part of the variance in Δ triacylglycerol and Δ carbohydrate (15.8% and 18.4% respectively, Table 3).

**Table 3:** Summary of multiple regression analyses using fatty acid food web markers in oysters as explanatory variables and triacylglycerol and carbohydrate (measured on 6 April and Δ) as response variables.

Response variables	Explanatory variables	Parameter estimates	SE	Partial r^2^	∑ r^2^	AICC	p value
√Triacylglycerol	Intercept	3.902	0.077			81.5	<0.001
	16∶1/16∶0	2.851	0.650	0.229	0.229	58.1	<0.001
	20∶5n-3	0.228	0.079	0.054	0.283	53.1	0.005
	18∶2+18∶3	0.384	0.155	0.025	0.308	51.8	0.015
	20∶5n-3×(18∶2+18∶3)	−0.119	0.063	0.024	0.332	50.7	0.062
	PUFA/SFA	−1.131	0.638	0.022	0.355	49.7	0.080
√Carbohydrate	Intercept	11.738	0.198			235.4	<0.001
	20∶5n-3	0.981	0.125	0.289	0.289	204.2	<0.001
	PUFA/SFA	−3.030	1.106	0.099	0.388	191.6	0.007
	20∶5n-3^2^	0.070	0.035	0.025	0.413	189.7	0.050
Log Δ triacylglycerol	Intercept	0.523	0.061			13.0	<0.001
	20∶5n-3	0.142	0.035	0.158	0.157	−0.7	<0.001
Log Δ carbohydrate	Intercept	0.340	0.041			−52.7	<0.001
	20∶5n-3	0.110	0.025	0.184	0.184	−69.5	<0.001
	18∶2+18∶3	−0.155	0.064	0.047	0.231	−72.8	0.018
	16∶1/16∶0	−0.649	0.336	0.031	0.262	−74.4	0.057

All variables were measured in oysters sampled in Thau Mediterranean lagoon.

## Discussion

Here we provide a large-scale high-resolution spatial representation of a disease mortality event affecting an economically important marine species, the oyster *Crassostrea gigas*. The spatial and temporal dynamics of disease-related mortality of oysters was analysed in relation with host energetic reserves and quality of food resources estimated by means of food web fatty acid markers.

### Spatial and Temporal Dynamics of Disease Mortality

Transmission of OsHV-1 within an oyster population occurs when susceptible hosts encounter infectious particles shed in the environment by neighboring infected individuals [36], [44], [45]. In our study, disease mortality of oysters showed strong spatial and temporal dependence, reflecting the epidemic process of local transmission [23]. Survival of oysters was lower within the bivalve farming area than outside it. Although the study design was not optimal to investigate survival time of oysters outside the farming areas, it appeared that the disease outbreak started within the bivalve farms. This result agrees well with the fact that aquaculture, which provides high-density populations of susceptible hosts, offers ideal conditions for disease epizootics [25], [46].

The disease affecting the oysters likely spread outside of bivalve farms via water currents. Indeed, marine parasites can be transported over considerable distances by seawater movement [25], [46], [47]. The ability of OsHV-1 to persist in seawater, even briefly, may be what allows the pathogen to spread to the whole lagoon. In a previous study, we showed that mortality of oysters varies between and within farming sites in a way that was consistent with the hydrodynamic regime and connectivity among areas where infected oysters were present [7]. For instance, mortality of oysters maintained in Thau lagoon but outside the farming area coincided with relatively strong currents coming from the farming area where mortality was occurring and OsHV-1 DNA was detected. Recently, the role of physical factors influencing infection prevalence of *Haplosporidium nelsoni*, causative agent of MSX disease in the eastern oyster *Crassostrea virginica*, was investigated by means of high-resolution hydro-dynamical model in Delaware Bay, USA [47]. These authors showed that spatial and temporal dynamics of *H. nelsoni* infection are related to seawater currents: infection prevalence at up bay locations corresponds to periods of enhanced cross-bay and up bay transport. Also, the spread of disease in aquaculture fish species implicate hydrodynamic regime, currents and the proximity of infected farms in the spread of diseases [48]–[52]. These findings are supported by modelling and have led to the adoption of management practices integrating the tidal excursion [52]. In our study, it is likely that the high survival of oysters observed at the south-eastern extremity of the lagoon reflects the influence of the Sète channel which connects the lagoon to the Mediteranean Sea [53], where no oyster mortality occurs [7].

Disease and related mortalities of oysters spread very rapidly at the lagoon scale, likely reflecting that the sentinel oysters were young, naïve and not selected for resistance. Indeed, susceptibility of oysters to OsHV-1 decreases with age of oysters, with past exposure to the pathogen, and with selection of resistant individuals [7], . Also, the rapid spread of the disease and related mortalities of oysters is probably typical of this Mediterranean lagoon, where mortality rates of oysters caused by OsHV-1 are the highest compared to other French Atlantic areas [57]. Spatiotemporal studies of disease mortality in oysters are currently underway in other observation areas in France.

Risk of oyster mortality was positively correlated with detection of OsHV-1 DNA in oyster tissues, which agrees well with the idea that OsHV-1 is a causal factor of mass mortality [7−9], [11], [44], [56], . It is, however, noteworthy that detection of OsHV-1 DNA in great amount in oysters tissues (on 16 April, the quantity of the viral DNA had increased up to 9.0•10^8^ copy number mg^−1^ wet tissue of oysters, regardless of areas) does not necessarily lead to high mortality rate, as observed in oysters maintained free of mortality in the south-easternmost part of the lagoon (present study), in resistant animals [56] or in oysters cemented onto ropes at low density [7]. These previous studies showed that OsHV-1 positive oysters which exhibit no or low mortality clear the viral DNA from their tissue more rapidly than those which suffer high mortality. It seems that the capacity to rapidly eliminate the viral DNA is linked with virus resistance and survival in oysters.

### Energetic Reserves and Survival

Our study provides evidence that, under natural conditions, the risk of disease mortality in oysters exposed to the herpesvirus OsHV-1 decreases with increasing mobilization of energetic reserves during the infection period. It was previously suggested that oysters with high energy reserves may be less affected by pathogen infections than those with low levels [7]. For instance, higher levels of triacylglycerol in oysters cemented to ropes coincided with lower mortality and limited proliferation of OsHV-1 compared with oysters held in Australian baskets [7]. Interestingly, the present study reveals that mobilization of energetic reserves in oysters during the period of infection is a better predictor of the risk of disease mortality than the initial level of reserves. It is, however, noteworthy that these two variables are positively correlated, so that their effects on oyster mortality risk are somewhat confounded. Also, it cannot be ascertained whether the decrease in energy reserves contributed to the mass mortality as the effect of other unmeasured confounding factors cannot be ruled out. The correlative approach used in this study prevents us from establishing causal relationship.

The use of energetic reserves during the period of infection may fuel oyster immune response. Therefore, higher mobilization of energetic reserves may lead to a stronger immune response, which increases the survival time of oysters. In bivalves, immunity consists of innate processes, including various serologically active molecules and of phagocytosis accompanied by production of oxygen metabolites and the release of lysosomal enzymes [61]. Recent studies show that bivalves exposed to pathogenic bacteria allocate a part of their energy from feeding and energetic reserves to sustain immune, antioxidant and cytoprotection processes [62], [63]. For example, oyster larvae exposed to pathogenic *Vibrio coralliilyticus* had lower triacylglycerol and protein content, the two main energetic reserves in bivalve larvae, and higher abundance of transcripts of antioxidant enzymes and immune-related proteins than uninfected larvae [62]. However, these authors also reported that feeding activity in challenged oysters decreased markedly compared with that of control animals, which is symptomatic of pathogen infection in bivalves [62], [64]. Therefore, the decrease in energetic reserves observed during disease outbreaks in the present study may reflect not only the cost of immune response, but also a concomitant reduction in food acquisition.

### Energetic Reserves and Food Quality

The local environment causes marked changes in energetic reserves of oysters and their utilisation during the infection period. The observed changes in energetic condition of oysters partly reflect natural variation in food quality. For instance, energetic reserves of oysters were positively correlated with 16-1n7/16∶0 and 20∶5n-3, two diatom markers commonly used in trophic ecology [34], [35]. This result agrees well with previous studies showing that diatoms are the main food source sustaining oyster growth and development in Thau lagoon, whereas terrestrial, animal (small zooplankton such as ciliate protists and tintinnids) and bacterial organic matter are secondary food sources that mostly contribute to the diet of oysters during non-bloom periods [17], [65]. Therefore, food quality explains a significant part of the variance in energetic reserves of oysters and their utilisations, which in turn is negatively correlated with the risk of disease mortality.

Although we specifically tested for the effect of food quality on energetic reserves of oysters, we cannot rule out the effect of other factors such as food availability. Several studies have found that shellfish farms have a large impact on plankton communities and biomass. For example, a study conducted in 1991–92 reported that the presence of shellfish farms led to a ∼40% deficit in chlorophyll *a* in the western part of the Thau lagoon [66]. It is therefore likely that oysters held outside of the farming area benefit from more food than those within it, thus enhancing their energetic reserves and their disease resistance.

## Conclusion

This study shows that local differences in the use of energetic reserves of oysters, partly driven by variation in food quality, play a significant role in the spatial and temporal dynamics of disease mortality. In particular, the relative contribution of diatoms to the diet of oysters correlates with their energetic reserves and their utilisation during the infection period which decreases the risk of disease mortality. Therefore, energetic status and food quality could have major implications for host-pathogen dynamics in marine ecosystems. Our study provides a better understanding of the factors that contribute to transmission of disease mortalities in the aquatic environment, which is necessary to build realistic predictive modelling of disease mortalities. Such models are needed as tools to test disease control scenarios that could mitigate the impact of disease mortalities and could potentially be adapted to other epizootic events that may occur in the near future.

## Supporting Information

File S1 **Laboratory analyses.**
(PDF)

File S2 **Spatial and temporal dynamics of oyster mortality (kriged maps and variogram).**
(PDF)

File S3 **Spatial distribution of OsHV-1 DNA in oysters.**
(PDF)

File S4 **Fatty acid food web markers in oysters (kriged maps and result description).**
(PDF)
