# Supplementary material for: Spatial and Temporal Dynamics of Mass Mortalities in Oysters Is Influenced by Energetic Reserves and Food Quality
Source: PLoS One. 2014 Feb 14;9(2):e88469. doi: 10.1371/journal.pone.0088469 (PMC3925110; doi:10.1371/journal.pone.0088469)
Supplement: File S1 — Laboratory analyses. (PDF) [file pone.0088469.s001.pdf]

Electronic Supplementary Material

S1. LABORATORY ANALYSES

OsHV-1 DNA

A sub-sample of ground oyster tissues (50-100 mg of fine powder) was diluted in artificial seawater (1:4 m/v). Total DNA was then extracted using a QIAgen QIAamp tissue mini kit according to the manufacturer's protocol. The extracted DNA was stored at -20°C prior to pathogen detection and quantification by qPCR.

The detection and quantification of OsHV-1 DNA was carried out using a previously published real-time PCR protocol [1]. Briefly, this protocol used SYBR® Green chemistry with specific DPFor/DPRev primers targeting the region of the OsHV-1 genome predicted to encode a DNA polymerase catalytic subunit [2]. Amplification reactions were performed using an Mx30005P real-time PCR thermocycler sequence detector (Stratagene, La Jolla, CA, USA) with 96-microwell plates [1]. Data are expressed as the number of experimental units (pearl nets containing the sampled oysters) in which DNA of OsHV-1 was detected.

Energetic reserves

For lipid analyses, powder aliquots (150 mg) were placed in amber glass vials filled with 3 ml chloroform-methanol (2:1 v/v) for further extraction following Folch [3]. Extracts were concentrated and applied to SIII Chromarods (Iatron laboratories, Tokyo, Japan). Lipid classes were separated in a stepwise procedure using developing solvents of increasing polarity [4] to separate aliphatic hydrocarbons, sterol and wax esters, ketones, triacylglycerols, free fatty acids, free fatty alcohol, free sterols, diacylglycerols, acetone mobile polar lipids and phospholipids. Between each development, the Chromarods were partially scanned by flame ionization detection (FID) on an Iatroscan MK-VI (Iatron

Laboratories, Tokyo, Japan). For the purpose of this study, only triacylglycerol in whole tissues of oysters is presented.

For carbohydrate analyses, powder aliquots (100 mg) were placed in Eppendorf tubes containing 1.5 mL nanopure water, homogenised for ~30 s with a T10 basic ultra Turrax (IKA, Germany). A subsample of the diluted powder was mixed with a phenol solution (5% m/v) and 2.5 mL H_2SO_4 and incubated for twenty minutes [5]. This subsample was then placed in a spectrophotometer and its absorbance measured at 490 and 600 nm. Total carbohydrate concentration was calculated using a standard calibration curve.

Triacylglycerol and carbohydrate are expressed in mg g^{-1} dry mass of tissues. The relative variation of these energetic reserves was calculated between 6 and 16 April according to the following formula:

$\Delta_{reserve} = \frac{(X_{06April} - X_{16April})}{X_{06April}}$, where *X* represents triacylglycerol or carbohydrate.

Fatty acids

An aliquot of the lipid extraction was evaporated to dryness and lipids recovered with three 500 mL washings of $CHCl_3$-MeOH (98:2 v/v). The lipids were placed at the top of a silica gel microcolumn (30 × 5 mm internal diameter; Kieselgel; 70–230 mesh [Merck, Lyon, France]; previously heated to 450°C and deactivated with 5% water). The neutral lipids were eluted with 10 mL $CHCl_3$-MeOH mixture (98:2 v/v). The polar lipids were recovered with 15 mL methanol. A known amount of 23:0 fatty acid was added as an internal standard. Lipids were transesterified with 10 wt% boron trifluoride-methanol [6] and analysed according to the method described by Marty et al. [7]. The fatty acid methyl esters (FAME) were analysed in a gas chromatograph with an on-column injector, a DB-Wax (30 m × 0.25 mm; 0.25 μm film thickness) capillary column and a flame ionization detector. Hydrogen was used as the carrier gas. Only the fatty acids in the neutral lipids of the animals are presented here, since neutral

lipids reflect the fatty acid profiles of food consumed and could reveal information about trophic sources, whereas fatty acids in the polar lipids are less sensitive to dietary changes [8].

References

1. Pepin JF, Riou A, Renault T (2008) Rapid and sensitive detection of ostreid herpesvirus 1 in oyster samples by real-time PCR. J Virol Methods 149: 269-276. doi: 10.1016/j.jviromet.2008.01.022
2. Webb SC, Fidler A, Renault T (2007) Primers for PCR-based detection of ostreid herpes virus-1 (OsHV-1): Application in a survey of New Zealand molluscs. Aquaculture 272: 126-139. doi: 10.1016/j.aquaculture.2007.07.224
3. Folch J, Lees M, Sloane-Stanlez GH (1957) A simple method for the isolation and purification of total lipids from animal tissues. J Biol Chem 226: 497-509. doi: not found
4. Parrish CC (1999) Determination of total lipid, lipid classes, and fatty acids in aquatic samples. In: Arts MT, Wainman BC, editors. Lipids in Freshwater Ecosystems. New York: Springer-Verlag. pp. 5-20.
5. DuBois M, Gilles KA, Hamilton JK, Rebers PA, Smith F (1956) Colorimetric method for determination of sugars and related substances. Anal Chem 28: 350-356. doi: 10.1021/ac60111a017
6. Metcalfe LD, Schmitz AA (1961) The rapid preparation of fatty acid esters for gas chromatographic analysis. Anal Chem 33: 363-364. doi: 10.1021/ac60171a016
7. Marty Y, Delaunay F, Moal J, Samain J-F (1992) Changes in the fatty acid composition of *Pecten maximus* (L.) during larval development. J Exp Mar Biol Ecol 163: 221-234. doi: 10.1016/0022-0981(92)90051-B
8. Dalsgaard J, St. John M, Kattner G, Muller-Navarra D, Hagen W (2003) Fatty acid trophic markers in the pelagic marine environment. Adv Mar Biol 46: 225-340. doi: 10.1016/S0065-2881(03)46005-7
