# Supplementary material for: Spatial and Temporal Dynamics of Mass Mortalities in Oysters Is Influenced by Energetic Reserves and Food Quality
Source: PLoS One. 2014 Feb 14;9(2):e88469. doi: 10.1371/journal.pone.0088469 (PMC3925110; doi:10.1371/journal.pone.0088469)
Supplement: File S2 — Spatial and temporal dynamics of oyster mortality (kriged maps and variogram). (PDF) [file pone.0088469.s002.pdf]

Electronic Supplementary Material

S2. SPATIAL AND TEMPORAL DYNAMIC OF OYSTER MORTALITY

Figure S2.1. Kriged maps of cumulative mortality of oysters in the Mediterranean Thau lagoon as a function of sampling times. Black points represent sampling sites and areas with grey boxes symbolize bivalve farms.

Figure S2.2. Variogram of the mean survival time of oysters in Mediterranean Thau lagoon. Grey points represent mean variance between pairs of distance points (numbers of pairs used are indicated above each point), red line represent the model variogram. Nugget represents sum of micro-scale variations or measurement errors. Sill is the semivariance value at which the variogram levels off. Range is the distance at which mean survival time between sampling points no longer correlate, or the distance at which the difference between the variogram and the sill becomes negligible. Here, the range was 1.5 km, the distance at which the semivariance level off. This variogram model was used for producing kriged map of the mean survival time of oysters (Figure 2a).

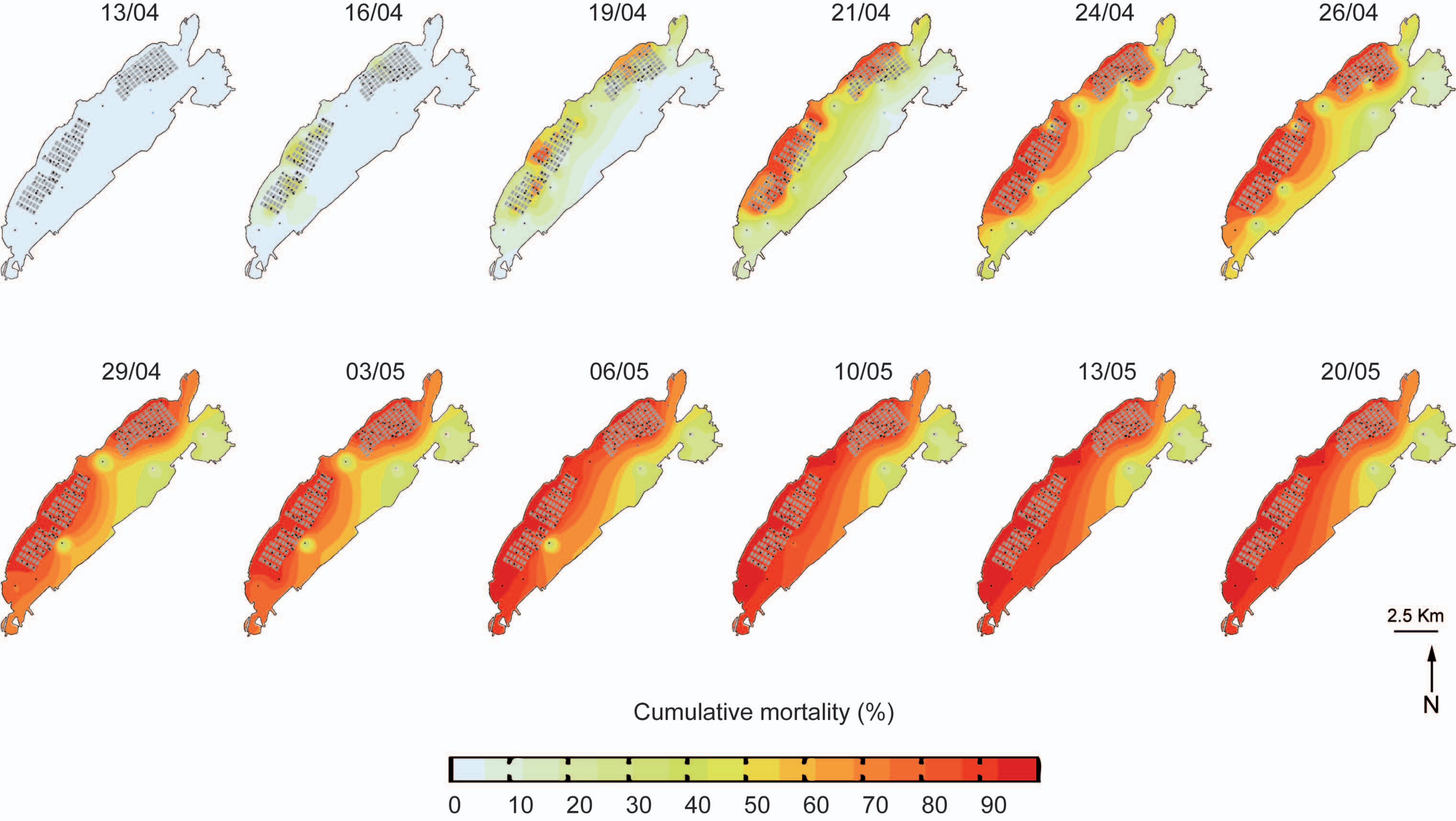
13/04
16/04
19/04
21/04
24/04
26/04
29/04
03/05
06/05
10/05
13/05
20/05
2.5 Km
N
Cumulative mortality (%)
0
10
20
30
40
50
60
70
80
90

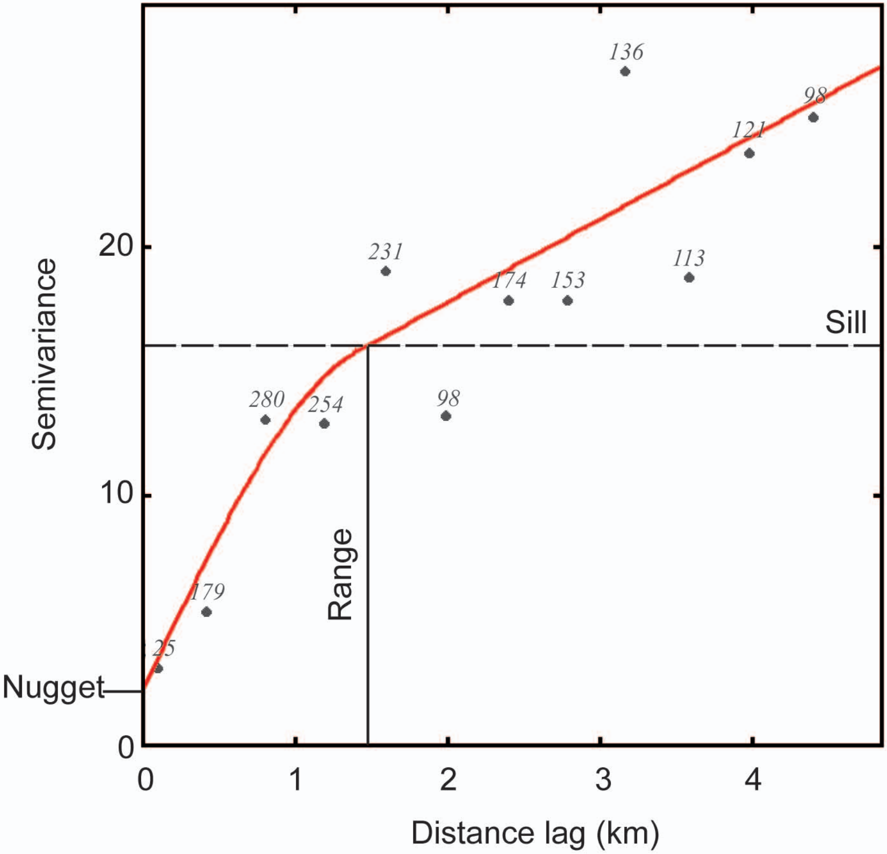

Figure S2.2. - ESM
