# Supplementary material for: Spatial and Temporal Dynamics of Mass Mortalities in Oysters Is Influenced by Energetic Reserves and Food Quality
Source: PLoS One. 2014 Feb 14;9(2):e88469. doi: 10.1371/journal.pone.0088469 (PMC3925110; doi:10.1371/journal.pone.0088469)

Electronic Supplementary Material

S3. DETECTION OF OsHV-1 DNA

Figure S3.1. Spatial distribution of OsHV-1 DNA in oysters sampled on 06 April 2011 in Thau lagoon. Areas with grey boxes symbolize bivalve farms.

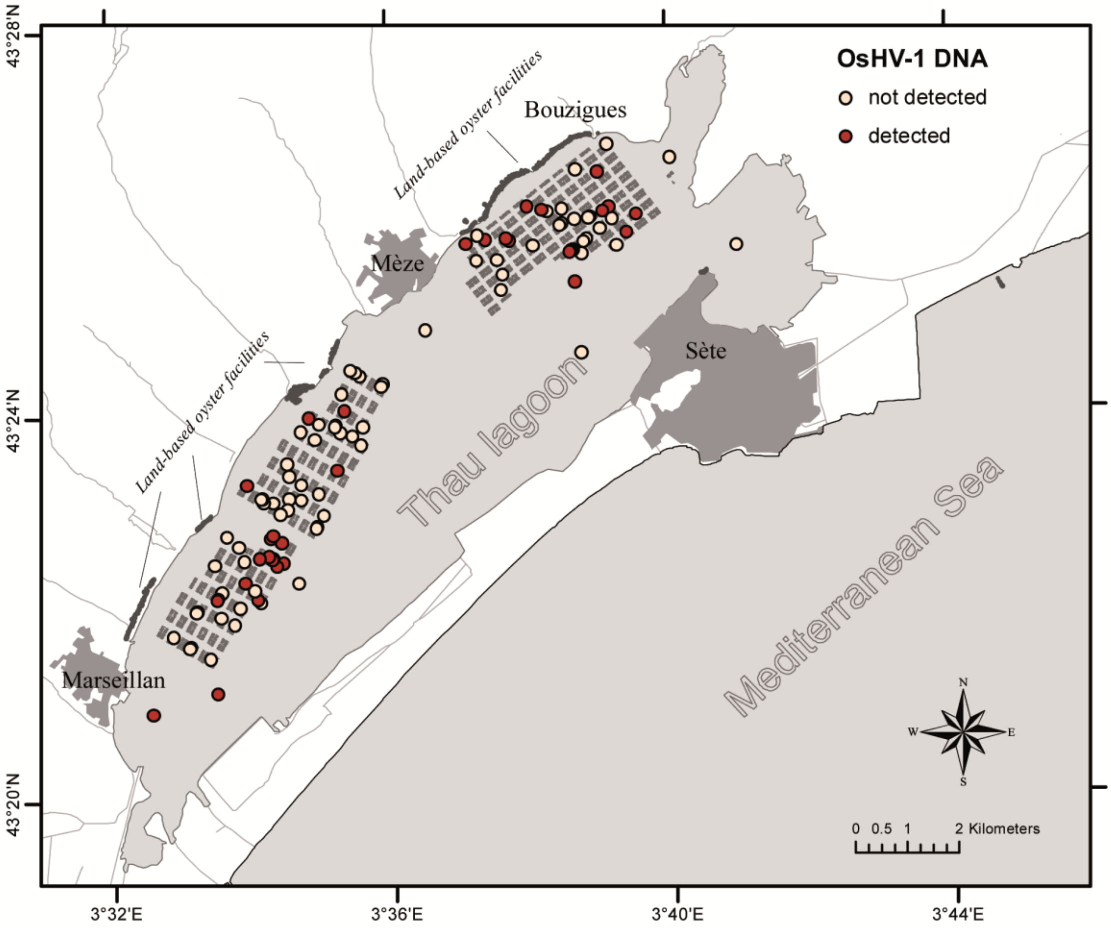

Supplement: File S3 — Spatial distribution of OsHV-1 DNA in oysters. (PDF) [file pone.0088469.s003.pdf]
