# Supplementary material for: Spatial and Temporal Dynamics of Mass Mortalities in Oysters Is Influenced by Energetic Reserves and Food Quality
Source: PLoS One. 2014 Feb 14;9(2):e88469. doi: 10.1371/journal.pone.0088469 (PMC3925110; doi:10.1371/journal.pone.0088469)
Supplement: File S4 — Fatty acid food web markers in oysters (kriged maps and result description). (PDF) [file pone.0088469.s004.pdf]

Electronic Supplementary Material

S4. SPATIAL ANALYSIS OF FATTY ACID FOOD WEB MARKERS

The two diatom markers, 16:1n-7/16:0 and 20:5n-3, varied by 4.2 and 1.6 times respectively, which suggest that the contribution of diatom to the diet of oysters varied markedly within the lagoon. Based on these markers, it seemed that oysters held in the farming area of Marseillan were less exposed to diatoms compared to animals held in other areas (figure S3*a*, *b*, see insets).

The sum of 18:2n−6 and 18:3n−3, which is generally considered as a marker of terrestrial inputs, was the lowest at Bouzigues where 20:5n-3 attained the highest values (figure S3*c*). Interestingly, the highest values of 18:2n-6+18:3n-3 (5.6%) was observed at the south westernmost side of the lagoon, close from the Marseillan harbour, in the shallowest waters.

The ratio of 18:1n-9 and 18:1n-7, which is used as a marker of animal tissues, varied from as low as 0.1 to 1.5 according to sampling sites (figure S3*d*). Values of this ratio were the highest at Marseillan where diatom markers showed the lowest levels, intermediate at Mèze, and the lowest at Bouzigues and outside of the farming areas. The ratio of 18:1n-9 and 18:1n-7 was negatively correlated with 20:5n-3 (r^2=0.563, $p<0.001$, n=98), suggesting that animal and diatom contributions to the diet of oysters were opposite.

The ratio of PUFA/SFA, which is an indicator of freshness, varied from 1.1 to 2.4 with no clear pattern within the lagoon (figure S3*d*). It is however noteworthy that PUFA/SFA was positively correlated with 20:5n-3 (r^2=0.506, $p<0.001$) and negatively correlated with 18:1n-9/18:1n-7 (r^2=0.476, $p<0.001$).

Finally, the sum of iso- and anteiso-branched chain fatty acids and unbranched 15:0 and 17:0, which reflect the contribution of bacteria to the organic matter, varied locally from 1.2% to 2.8%. The highest value was observed at Mèze, in a shallow area close from the shore characterized by high density of land-based oyster farms (see figure 1 and S3*e*).

Figure S4.1. Kriged maps of fatty acid food web markers in oysters in the Mediterranean Thau lagoon measured on 06 April. Black points represent sampling sites, areas with grey boxes symbolize individual bivalve farms and dashed rectangles correspond to the three farming areas. Inset graphs represent fatty acid food web markers of oysters as a function of time and areas in the Thau lagoon. Letters indicate significant differences. Data are means ± SD.

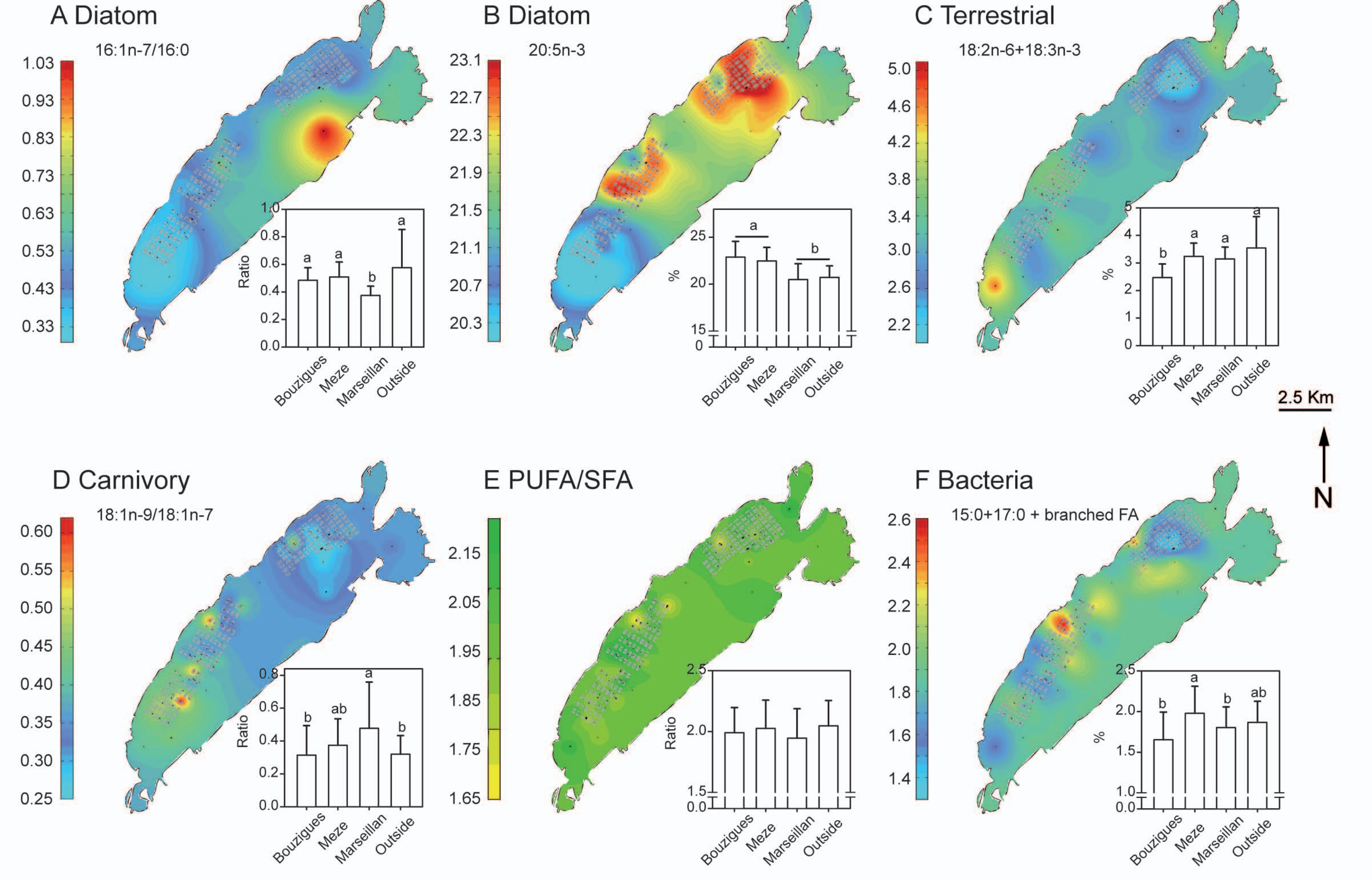

Figure S4.1. - ESM
